# Granule cell excitability mediates gamma and beta oscillations in a model of the dendrodendritic microcircuit

**DOI:** 10.1186/1471-2202-16-S1-P118

**Published:** 2015-12-18

**Authors:** Boleslaw L Osinski, Leslie M Kay

**Affiliations:** 1 Biophysical Sciences, University of Chicago, Chicago, IL 60637, USA; 2 Institute for Mind and Biology, University of Chicago, Chicago, IL 60637, USA; 3 Department of Psychology, University of Chicago, Chicago, IL 60637, USA

## 

Odors evoke gamma (60 - 100 Hz) and beta (20 - 30 Hz) oscillations in the local field potential (LFP) of the rat olfactory bulb (OB). These oscillations arise from activity in the dendrodendritic microcircuit between excitatory mitral cells (MCs) and inhibitory granule cells (GCs) [1]. When cortical feedback inputs to the OB are blocked, beta oscillations are extinguished while gamma oscillations persist [2]. Much of this cortical feedback targets inhibitory interneurons in the GC layer and regulates the excitability of GCs [3], which suggests a causal link between the emergence of beta oscillations and the GC excitability. We investigate the effect that GC excitability has on network oscillations in a biophysical model of the MC-GC dendrodendritic network with graded inhibition. When GC excitability is low, there is transient activation of NMDAR channels by AMPARs, which produces fast inhibitory pulses in the gamma frequency range. When GC excitability is increased, the activation of NMDARs and other VDCCs is prolonged, allowing the slow decay time constants of these channels to drive beta frequency oscillations. The power of the beta oscillation peaks when inhibitory and excitatory currents onto MCs are balanced, which could explain the relationship between beta power and odor volatility measured experimentally [4].
